# Meningeal null cell lymphoma causing diffuse pachymeningeal contrast enhancement in a dog

**DOI:** 10.1111/jsap.13810

**Published:** 2024-12-10

**Authors:** M. Madden, N. Israeliantz, A. Malbon, C. Piccinelli, K. Marioni‐Henry, T. Schwarz, A. Suñol

**Affiliations:** ^1^ Hospital for Small Animals, Royal (Dick) School of Veterinary Studies University of Edinburgh Edinburgh UK; ^2^ IDEXX Laboratories Ltd Wetherby UK; ^3^ Fitzpatrick Referrals Godalming UK; ^4^ Hospital Veterinaria del Mar, IVC Evidensia Barcelona Spain; ^5^ Hospital Clinico Veterinario Universidad Europea Madrid Spain

## Abstract

Diffuse pachymeningeal contrast enhancement is an uncommon imaging finding in dogs and current understanding of its aetiologies in veterinary medicine is limited. A 2‐year‐old female neutered Pug presented with chronic progressive vestibular signs, facial nerve paralysis, obtundation and episodic decerebellate rigidity. A magnetic resonance imaging study of the head revealed diffuse pachymeningeal thickening and contrast enhancement involving the caudal fossa and falx cerebri. Cerebrospinal fluid analysis revealed marked neutrophilic pleocytosis. Infectious disease testing was negative and a tentative diagnosis of intracranial idiopathic hypertrophic pachymeningitis was made. Immunosuppressive treatment resulted in a short period of clinical improvement. The patient subsequently suffered multiple relapses which failed to respond to alternative immunosuppressive strategies, necessitating euthanasia. Histopathological findings supported a meningeal null cell lymphoma. While rare, neoplastic causes of diffuse pachymeningeal thickening and contrast enhancement should be considered.

## INTRODUCTION

Patterns of meningeal contrast enhancement on magnetic resonance imaging (MRI) studies can be classified as pachymeningeal (involving the dura mater and inner layer of the periosteum of the skull) or leptomeningeal (involving the arachnoid and pia mater) (d'Anjou et al., 2012). Discriminating between pachymeningeal and leptomeningeal contrast enhancement relies on the detection of contrast within the pial vasculature of the cerebral sulci (d'Anjou et al., 2012). While a degree of meningeal contrast enhancement has been reported as a normal MRI finding in dogs (Joslyn et al., 2011), it should be considered pathological if it is present on multiple adjacent slices, or exhibits an increased thickness, signal intensity or nodular appearance (Kirmi et al., 2009; Patel & Kirmi, 2009). In terms of distribution, it can be focal (limited to one region) or diffuse (spread over multiple regions). Focal pachymeningeal contrast enhancement tends to be associated with an adjacent disease process, for example, the dural tail associated with a meningioma, or an infectious pachymeningitis associated with intracranial extension of otitis media and interna (Graham et al., 1998; Mellema et al., 2002), whereas diffuse pachymeningeal contrast enhancement is often reported as an independent finding (Antony et al., 2015). Diffuse pachymeningeal contrast enhancement is rarely reported as an MRI finding in dogs (Table 1).

**Table 1:** Causes of diffuse pachymeningeal contrast enhancement in dogs

Category	Disease
Immune‐mediated	Steroid‐responsive meningitis arteritis with intracranial involvement (Tipold & Schatzberg, 2010)
Idiopathic	Idiopathic hypertrophic pachymeningitis (Roynard et al., 2012)
Iatrogenic	Craniotomy (Chow et al., 2015) Intracranial hypotension following surgery (Cloquell et al., 2021)
Neoplastic	Lymphoma (d'Anjou et al., 2012; Mellema et al., 2002) B‐cell lymphoblastic leukaemia with meningeal metastases (Mellema et al., 2002; Vernau et al., 2000) Meningeal granular cell tumour (Anwer et al., 2013; Mishra et al., 2012) Meningioma *en plaque* (Mai, 2018)

Causes of diffuse pachymeningeal contrast enhancement taken from published veterinary literature containing consistent advanced imaging findings

In this case report, we describe the clinical and MRI features of a dog with meningeal null cell lymphoma with a diffuse pachymeningeal distribution. Null cell lymphoma is a rare subtype of lymphoma in which neoplastic cells do not express routine B‐ or T‐lymphocyte markers on flow cytometry or immunohistochemistry (Jacobsen, 2006), representing less than 5% of all canine lymphomas (Ponce et al., 2010; Vail et al., 2019). According to a literature search [MEDLINE/Pubmed and ScienceDirect (performed on 24 October 2024)] using relevant keywords (“null cell lymphoma,” “dog” and “canine”), no cases of meningeal null cell lymphoma have been reported to date.

## CASE DESCRIPTION

**FIG 1:**
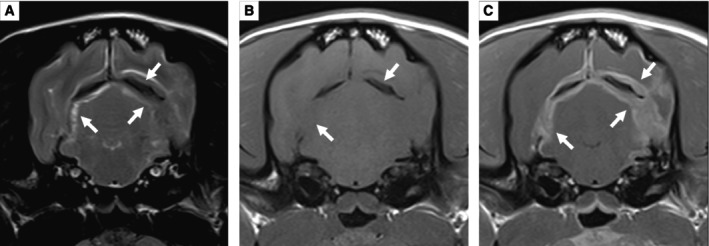
T2‐weighted (A), T1‐weighted (B) and T1‐weighted following contrast administration (C) transverse images at the level of the rostral medulla oblongata and tentorium cerebelli. Pronounced and diffuse pachymeningeal thickening involving the tentorium cerebelli and falx cerebri is visible (arrows), appearing hyperintense on T2‐W images, iso‐ to hypointense on T1‐W images and strongly contrast enhancing following gadolinium administration.

**FIG 2:**
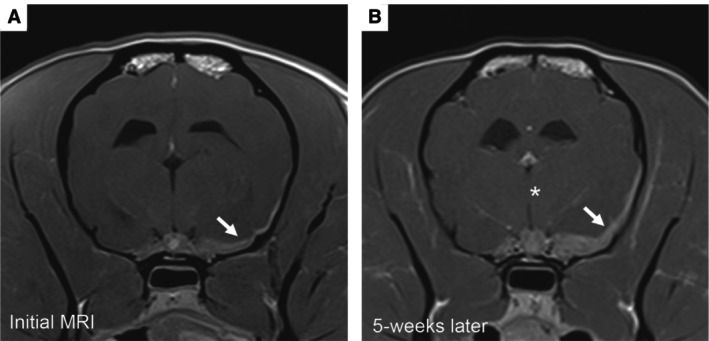
T1‐weighted transverse images after contrast administration at the level of the hypophysis at the time of the initial MRI study (A) and repeat MRI study 5 weeks later (B). Pachymeningeal thickening surrounding the left temporal lobe is more pronounced in B (arrows). Mild dextrad midline shift of the brain parenchyma is also present (star).

A 2‐year‐old 7.8 kg female neutered Pug presented with a 2‐week history of progressive vestibular signs, facial nerve paralysis, altered mentation and episodic decerebellate rigidity. Complete blood count, serum biochemistry and electrolyte testing performed by the primary care veterinarian were unremarkable. Physical examination was unremarkable. Neurological examination revealed obtundation, left‐sided head tilt with low head carriage, vestibular ataxia affecting all four limbs (falling to the left) and a left‐sided facial nerve paralysis. Neurolocalisation was multifocal, including the left brainstem, cerebellum and left facial nerve. Differential diagnoses included auto‐immune (e.g., meningoencephalitis of unknown origin), infectious (e.g., otitis media with secondary bacterial meningoencephalitis) and neoplastic (e.g., glioma, lymphoma) disease. A MRI (Magneton Avanto 1.5 Tesla, Siemens) study of the head with contrast (gadoteric acid, 0.1 mmol/kg iv, Dotarem, Guerbet) revealed diffuse thickening of the pachymeninges involving the tentorium cerebelli, falx cerebri and left brainstem (Fig 1). Relative to the adjacent brain parenchyma, the pachymeninges were T2‐weighted and T1‐weighted hypointense centrally, with a T2‐weighted hyperintense, T1‐weighted isointense and strongly, homogeneously contrast enhancing periphery. No leptomeningeal or intraparenchymal involvement was observed. Cerebrospinal fluid (CSF) collected from the cerebellomedullary cistern revealed a total nucleated cell count of 268/μl (reference range 0 to 5/μl), red blood cells 99/μl (reference range 0/μl) and total protein 76 mg/dL (reference range <25 mg/dL) (Platt & Olby, 2013). Cytological examination revealed a marked neutrophilic pleocytosis (61% non‐degenerate neutrophils, 20% large monocytoid cells and 19% small lymphocytes). No infectious agents or atypical cells were observed. Bacterial, yeast, fungal and prolonged anaerobic cultures of the CSF and serum antibody titres against *Toxoplasma gondii* and *Neospora caninum* returned negative. An auto‐immune cause, that is, intracranial idiopathic hypertrophic pachymeningitis (iHPM) was suspected. The patient demonstrated a positive initial response to immunosuppressive treatment with corticosteroids (prednisolone, 2 mg/kg po q24h, Prednicare, Animal Care) and cytosine arabinoside [100 mg/m^2^ sc q12h (total dose 200 mg/m^2^), ARA‐cell, Accord Healthcare Limited]. However, the patient continued to experience a relapsing–remitting course of disease despite the implementation of alternative immunomodulatory medications (leflunomide, 3.3 mg/kg po q24h, Tillomed Laboratories Ltd) alongside immunosuppressive doses of corticosteroid medication. Due to the recurrent relapses, a repeat MRI was performed which showed progression of the pachymeningeal thickening (Fig 2). A surgical biopsy of the dura mater was offered but declined by the owners. Owing to the poor prognosis, the patient was humanely euthanised at this point, 5 weeks following the initial presentation.

**FIG 3:**
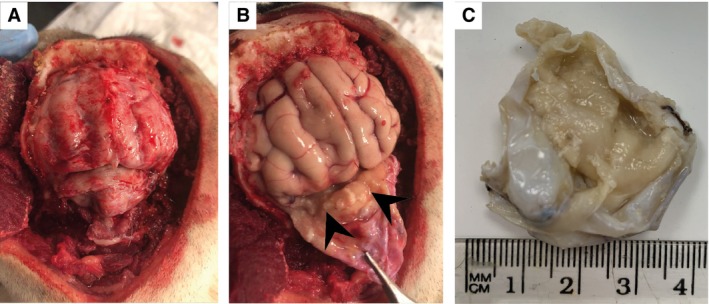
Gross post‐mortem images. (A) Dorsal view of the head. The frontal, parietal and occipital bones have been removed to reveal the brain in situ with the dura mater in tact. (B) Reflection of the dura mater revealing a yellow to pinkish granular texture to the internal surface and presence of a small volume of yellow, watery fluid (black arrowheads). (C) Dura mater following formalin fixation.

**FIG 4:**
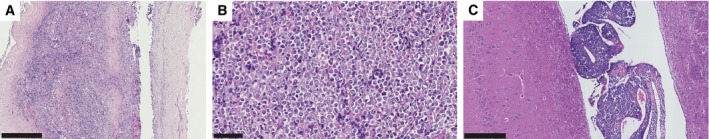
(A) Microscopic image of histological section of dura mater. Nuclei often exceeded twice the diameter of an erythrocyte and cells exhibited moderate anisokaryosis and anisocytosis, with mitotic figures greater than 10 per high powered field. Haematoxylin and eosin stain. Scale bar 250 μm. (B) Microscopic image of dura mater. Haematoxylin and eosin stain. Scale bar 50 μm. (C) Microscopic image of a histological section of the choroid plexus in a lateral ventricle. Haematoxylin and eosin stain. Scale bar 250 μm.

On post‐mortem examination of the cranial vault, the dura mater was found to be diffusely thickened, particularly in the region of the tentorium cerebelli (Fig 3). The inner surface of the dura mater exhibited a yellow to pinkish granular texture and enclosed a small volume of yellow, watery fluid **(**Fig 3B **)**. Gross examination of the brain, cranial vault and cranial nerves at the level of their attachments was unremarkable. Histological examination of the dura mater revealed an infiltrative and densely cellular neoplastic population of round cells with round to ovoid, to occasionally indented, hypochromatic nuclei, with coarsely stippled chromatin (Fig 4). Nuclei exhibited moderate anisokaryosis and anisocytosis, with mitotic figures greater than 10 per high powered field. The remainder of the dura mater exhibited sclerosis with lymphoplasmacytic infiltration. Sections through the brain at the level of the frontal cortex, hippocampus and cerebellum revealed variable extension of the neoplastic process into the subarachnoid space, with rare foci of cells invading through the pia mater into the parenchyma. The choroid plexuses of the lateral ventricles and the mesencephalic aqueduct were also expanded by neoplastic aggregates. These findings were consistent with a meningeal large cell lymphoma with mild subarachnoid involvement and minimal parenchymal extension, with a mild co‐existing inflammatory response. Polymerase chain reaction (PCR) antigen receptor rearrangement testing identified a clonal B‐cell receptor rearrangement. On immunohistochemistry, the neoplastic cells were negative for CD3, PAX5, CD79a, CD30 and Iba1. These findings were consistent with a diagnosis of a null cell phenotype.

## DISCUSSION

In this case report, we describe an unusual case of meningeal null cell lymphoma in a dog causing diffuse pachymeningeal contrast enhancement as the predominant imaging finding. Neoplastic causes of diffuse pachymeningeal contrast enhancement in dogs are rarely reported and, based on the author's literature search, this is the first confirmed report of meningeal null cell lymphoma in a dog to date.

Central nervous system (CNS) lymphoma is uncommon in dogs, representing 4% and 12% of primary and secondary intracranial tumours, respectively (Snyder et al., 2006, 2008). Intracranial CNS lymphoma can involve the brain parenchyma and/or meninges with focal, multifocal, diffuse and intravascular patterns described (Fonti et al., 2023). Canine meningeal lymphoma is scarcely reported in the veterinary literature (d'Anjou et al., 2012; Mellema et al., 2002). Primary meningeal lymphoma appears to be incredibly rare, with full post‐mortem and histological confirmation in only three dogs to date (Britt et al., 1984; Long et al., 2001; Rosin, 1982). Null cell lymphoma involving the CNS appears to be even rarer. In a study by LaRue et al. (2018), two of 18 dogs with CNS lymphoma exhibited a null cell phenotype; however, the precise anatomical distribution of lymphoma in these cases was not described. In humans, null cell lymphoma is most commonly encountered as a type of “anaplastic large cell lymphoma” (ALCL). Anaplastic large cell lymphoma is a form of large cell lymphoma in which neoplastic cells exhibit an anaplastic morphology and CD30+ immunoreactivity (Jacobsen, 2006). Most cases of ALCL are of T‐cell lineage, with a smaller proportion of null cell phenotype (Benharroch et al., 1998). Immunohistochemical testing in our patient was negative for CD30 making a diagnosis of ALCL unlikely. A major diagnostic limitation in the case we describe is the lack of ante‐mortem staging and full post‐mortem examination, precluding determination of a primary or metastatic meningeal null cell lymphoma.

In the case described, an initial diagnosis of intracranial iHPM was made based on the anatomical distribution and signal characteristics of the pachymeninges on MRI, in addition to normal physical examination findings, bloodwork parameters and negative infectious disease testing. Idiopathic HPM is an auto‐immune disease characterised by histological changes of diffuse hypertrophy and inflammation of the dura mater (Abrantes et al., 2020; Roynard et al., 2012). Common imaging features of intracranial iHPM in humans include diffuse pachymeningeal thickening with T2‐weighted hypointense signal (fibrosis) and peripheral contrast enhancement, predominantly involving the caudal falx cerebri and tentorium cerebelli producing a characteristic “Mercedes‐Benz sign” (Dash et al., 2015; Hahn et al., 2016) – comparable imaging findings have been reported in dogs with intracranial iHPM (Roynard et al., 2012). In humans, MRI findings of diffuse pachymeningeal thickening and contrast enhancement are not pathognomonic to intracranial iHPM and diagnostic investigations are tailored to exclude neoplastic, infectious and inflammatory causes. Ultimately, a dural biopsy is required to confirm a diagnosis of iHPM and exclude a neoplastic cause (Abrantes et al., 2020). Surgical resection of the dura mater can also provide a decompressive therapeutic effect (Williams et al., 2016). Due to the inherent risk associated with performing a dural biopsy/resection, it is not uncommon for human clinicians to start empirical treatment for iHPM (when investigations fail to identify an underlying cause) and reserve this procedure for cases which deteriorate or fail to respond appropriately (Dash et al., 2015; Hahn et al., 2016). In our patient, achieving an ante‐mortem diagnosis of lymphoma with dural biopsy would have provided valuable prognostic information and justification for more targeted treatment options such as systemic or intrathecal chemotherapy, or whole brain radiation.

In contrast to the MRI findings presented, the signalment of the patient (young, brachycephalic) and CSF analysis (marked non‐degenerate neutrophilic pleocytosis) deviated from reports of intracranial iHPM in dogs, which typically affects adult, large dolichocephalic breeds and produces a lymphocytic pleocytosis (Roynard et al., 2012). In veterinary patients, neutrophilic pleocytosis is most commonly reported with infectious (e.g., bacterial or viral meningoencephalitis) and immune‐mediated (e.g., steroid‐responsive meningitis‐arteritis, and less commonly, meningoencephalitis of unknown origin) inflammatory CNS disease (Granger et al., 2010; Levine & Cook, 2020; Tipold & Schatzberg, 2010). However, it has also been reported with CNS neoplasms including lymphoma and meningioma (Dickinson et al., 2006; Marioni‐Henry et al., 2008; Sisó et al., 2017). The mechanism through which CNS lymphoma could induce a neutrophilic pleocytosis is postulated to be due to neoplastic angioinvasion and secondary necrosis, which has been documented in a case of CNS T‐cell lymphoma in a human (Nakamura et al., 2020). The finding of such a marked neutrophilic pleocytosis in our patient was unusual and raised concern for a neoplastic process but given the lack of atypical lymphocytes on cytological examination, an auto‐immune cause remained prioritised at the time of diagnostic investigations.

In summary, diffuse pachymeningeal contrast enhancement is a rare imaging finding with diverse aetiologies in canine patients. This case underscores the diagnostic challenges posed by diffuse pachymeningeal contrast enhancement, emphasising the importance of thorough investigations including CSF analysis and extensive screening for systemic infectious, inflammatory or neoplastic diseases. We propose that dural biopsy should be considered when investigations fail to identify an underlying cause, there is progression of pachymeningeal thickening on advanced imaging, or the patient fails to respond appropriately to treatment.

## Author contributions

**M. Madden:** Conceptualization (equal); writing – original draft (equal); writing – review and editing (equal). **N. Israeliantz:** Writing – original draft (equal); writing – review and editing (equal). **A. Malbon:** Writing – original draft (equal); writing – review and editing (equal). **C. Piccinelli:** Writing – original draft (equal); writing – review and editing (equal). **K. Marioni‐Henry:** Writing – review and editing (equal). **T. Schwarz:** Writing – review and editing (equal). **A. Suñol:** Conceptualization (equal); supervision (equal); writing – review and editing (equal).

## Conflict of Interest

None of the authors of this article has a financial or personal relationship with other people or organisations that could inappropriately influence or bias the content of the paper.

## Data Availability

Data sharing is not applicable to this article as no datasets were generated or analysed during the current study. All relevant findings are presented in the article.
